# C2H2 Zinc Finger Proteins: The Largest but Poorly Explored Family of Higher Eukaryotic Transcription Factors

**Published:** 2017

**Authors:** A. A. Fedotova, A. N. Bonchuk, V. A. Mogila, P. G. Georgiev

**Affiliations:** Institute of Gene Biology, Russian Academy of Sciences, Vavilov Str., 34/5, Moscow, 119334, Russia

**Keywords:** architectural proteins, CTCF, KRAB domain, SCAN domain, transcription factors, ZAD

## Abstract

The emergence of whole-genome assays has initiated numerous genome-wide studies of transcription factor localizations at genomic regulatory elements (enhancers, promoters, silencers, and insulators), as well as facilitated the uncovering of some of the key principles of chromosomal organization. However, the proteins involved in the formation and maintenance of the chromosomal architecture and the organization of regulatory domains remain insufficiently studied. This review attempts to collate the available data on the abundant but still poorly understood family of proteins with clusters of the C2H2 zinc finger domains. One of the best known proteins of this family is a well conserved protein known as CTCF, which plays a key role in the establishment of the chromosomal architecture in vertebrates. The distinctive features of C2H2 zinc finger proteins include strong and specific binding to a long and unique DNA recognition target sequence and rapid expansion within various animal taxa during evolution. The reviewed data support a proposed model according to which many of the C2H2 proteins have functions that are similar to those of the CTCF in the organization of the chromatin architecture.

## INTRODUCTION

Recent genome-wide studies of intra- and interchromosomal interactions have revealed that the human, mouse, and *Drosophila *chromosomes are organized into large topologically associated domains (TADs) [1-4]. Long-distance interactions between promoters, enhancers, and silencers can occur within topological domains, which affect the regulation of gene expression [5, 6]. However, the mechanisms that underlie the organization and maintenance of the chromosomal architecture remain poorly understood [7]. It has been posited that there is a special class of architectural proteins whose inactivation significantly affects the distribution of inter- and intrachromosomal contacts [8, 9].

Vertebrates have a highly conserved transcription factor (TF), CTCF, which is considered to be the main architectural protein of chromosomes [10, 11]. CTCF, along with the cohesin complex, participates in the formation of topological domain boundaries and also maintains the long-distance interactions between the regulatory elements within the domains [12-14]. CTCF contains a cluster of C2H2 zinc finger domains, some of which are responsible for a highly specific binding of the protein to DNA. Proteins containing C2H2 zinc fingers (C2H2 proteins) emerged early during evolution and are found in many eukaryotes [15, 16]. Many of them are structurally similar to CTCF. C2H2 proteins could be divided into three groups [17]: 1) proteins with one, two, or several randomly distributed C2H2 domains; 2) proteins with three C2H2 domains organized into a C-terminal cluster; and 3) proteins with more than three C2H2 domains, forming one or more clusters. The best studied group includes conserved TFs with three C2H2 domains, with many of them playing a critical role in the regulation of gene expression in all higher eukaryotes [18, 19]. This review is devoted to the poorly studied TFs that contain more than three C2H2 domains.

## THE STRUCTURE AND FUNCTIONAL ROLE OF THE C2H2 DOMAIN

**Fig. 1:**
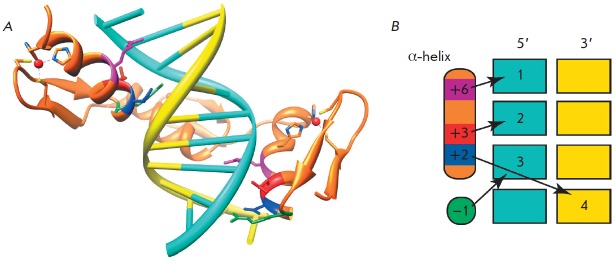
A model of the site-specific DNA recognition by C2H2 zinc finger domains. (A) The crystal structure of three zinc fingers of the Zif268 protein bound to DNA [20]. The amino acids involved in the site-specific DNA recognition are color-coded: –1 – green, +2 – blue, +3 – red, and +6 – purple. (B) A model of the site-specific DNA recognition by α-helical amino acids (adapted from [24]).

C2H2 zinc fingers (Cys2-His2) represent one of the most common domains found in the TFs of higher eukaryotes. The classical C2H2 domain of 28–30 aa includes a β-hairpin (antiparallel β-sheet consisting of two β-strands), followed by an α-helix, which form a left-handed ββα structure (*Fig. 1A*). The zinc finger structure is stabilized by the coordination of a zinc atom with two conserved cysteine residues at one end of the β sheet and with two conserved histidine residues at the α-helix C-terminus. The cysteine and histidine pairs are conserved, as well as the hydrophobic core forming the α-helix. The other amino acid residues in C2H2 domains are very variable.

One of the first structures to be determined was that of a complex of three tandem C2H2 domains of the mammalian Zif268 protein [20]. The three zinc fingers were found to form a semicircle located in the major DNA groove (*Fig. 1A*). Each of the three C2H2 domains binds to three or four DNA nucleotides via amino acids at the same α-helical positions (*Fig. 1B*): arginine at position –1, as well as amino acid residues at positions 2, 3, and 6. Biochemical and structural studies of the C2H2 domains confirmed the key role of the amino acids at these positions for the specific binding to DNA. According to the canonical model, the amino acids at positions 6, 3, and –1 are responsible for recognition of the first, second, and third nucleotides at the 5’-end, respectively, and the amino acid at position 2 recognizes the fourth nucleotide on the complementary strand (*Fig. 1B*).

Structural studies of C2H2 domains have revealed a new principle of DNA recognition. A distinctive feature of the C2H2 proteins is their specific binding to long (20–40 bp) DNA sequences, which distinguishes this class of proteins from the other TFs that usually recognize relatively short, degenerate DNA sequences. Typically, the tandem C2H2 domains involved in DNA recognition are separated by conserved sequences of 5 aa [21]. The existing algorithms can predict very accurately the binding sequence for a cluster of C2H2 domains and, conversely, to select C2H2 domain combinations that recognize a target DNA sequence [22, 23]. However, the interference between neighboring C2H2 domains in large clusters (more than three C2H2 domains) complicates an accurate prediction of the binding site [24].

**Fig. 2:**
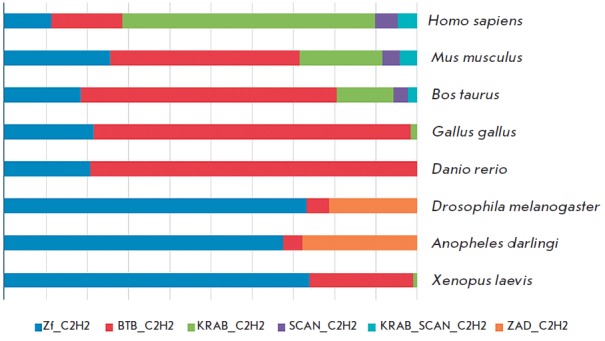
Relative abundance of different variants of C2H2 proteins in various higher eukaryotes: human (*Homo sapiens*), mouse (*Mus musculus*), wild bull (*Bos taurus*), chicken (*Gallus gallus*), zebrafish (*Danio rerio*), fruit fly (*Drosophila melanogaster*), anopheles mosquito (*Anopheles darlingi*), and frog (*Xenopus laevis*). Data were obtained from the Uniprot database.

In contrast to the invariant mechanism of the interactions between the C2H2 domains and DNA, contacts between the domains and proteins and RNAs form via various amino acid combinations, which has been detailed in other reviews [25, 26]. Typically, the C2H2 clusters are located in the middle or at the C-terminus of a protein. Most proteins that contain a C2H2 cluster in the middle position do not have other conserved domains. At the same time, proteins with a cluster at the C-terminus often contain additional N-terminal domains (*Fig. 2*). The KRAB and SCAN domains are typical of vertebrates, while the ZAD is typical of insects [27, 28]. A small group of C2H2 proteins has a conservative BTB/POZ domain at the N-terminus. This domain is often found in different classes of proteins. Therefore, we have excluded this group of C2H2 proteins from the present review. Comprehensive information on BTB-containing proteins is available in detailed reviews [29, 30].

## TRANSCRIPTION FACTORS CONTAINING ONLY A SINGLE CLUSTER OF C2H2 DOMAINS

The group of TFs containing only a single cluster of C2H2 domains includes the best studied and highly conserved CTCF protein (CCCTC-binding factor) [31] that was first described as a negative regulator of *myc *gene expression [32]. Later, a binding site for CTCF was found in an insulator located at the 5’-end of the chicken β-globin locus [33]. The CTCF binding sites are often located at the boundaries of chromosomal regions, which have different epigenetic statuses and transcriptional activity, as well as at the boundaries of the topologically associated domains (TADs) that spatially separate chromosomes into regions where interactions among regulatory elements occur [34-37].

**Fig. 3:**
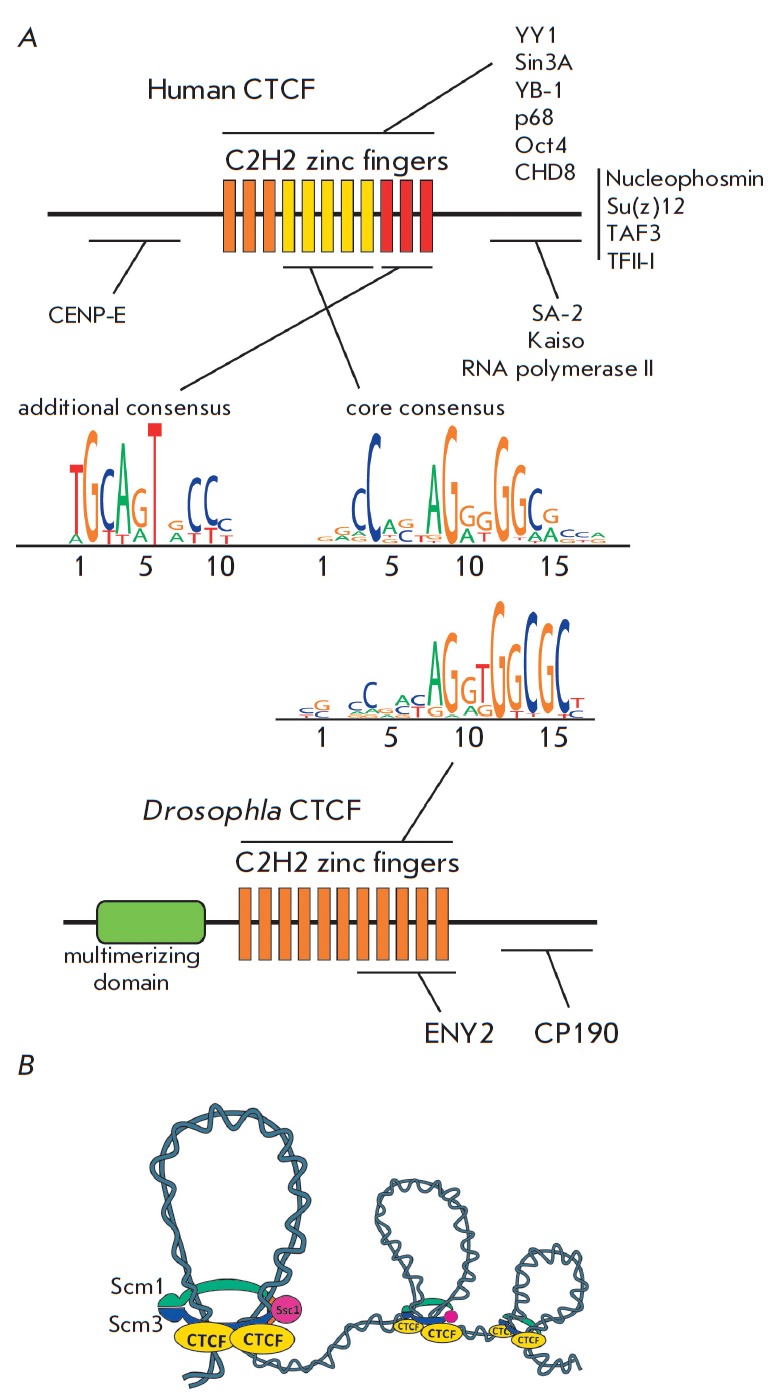
Comparison of the structures and properties of the *Drosophila *and human CTCF proteins. (A) The domain structures of the *Drosophila *and human CTCF proteins. The domains involved in the site-specific DNA recognition and the protein-protein interactions are represented by thin horizontal lines. *Drosophila* and human [46] CTCFs have similar consensus recognition sites. (B) The mechanism of the long-distance genomic interactions mediated by CTCF and cohesins.

CTCF is one of the few well conserved proteins that contain a cluster of C2H2 domains. A CTCF homolog in *Drosophila*, dCTCF, is also often found at the TAD boundaries and in insulators [38, 39]. In model transgenic systems, dCTCF maintains long-distance interactions between the reporter gene promoter and the GAL4 activator [40, 41]. There is a homodimerization domain at the N-terminus of dCTCF (*Fig. 3A*); probably, this domain is necessary for maintaining the long-distance interactions between remote dCTCF binding sites [42]. Attempts to find a similar dimerization domain in vertebrate CTCFs have not been successful. *In vitro *experiments have demonstrated that the C-terminal part of one CTCF molecule binds to the cluster of the C2H2 zinc finger domains of another CTCF molecule [43]. However, the specificity of this interaction has not been proven.

According to a generally accepted model, the cohesin complex required for homologous chromosome pairing during cell division [44] binds to CTCF and participates in the maintenance of the specific long-distance interactions between its sites in interphase chromosomes (*Fig. 3B*). The region interacting with one of the cohesin complex proteins was mapped to the C-terminal domain of human CTCF [44].

The binding of CTCF to DNA, which is conserved even between insects and mammals, has been thoroughly studied in many higher eukaryotes [15, 45]. The C2H2 domains 4 to 7 of CTCF (*Fig. 3A*) participate in the binding to a core consensus site [46, 47]. Approximately 20% of the sites contain a second 10 bp motif that associates with the C2H2 domains 9 to 11 [47, 48]. This second motif, separated by 5 or 6 bp from the first, is supposed to increase the stability of CTCF binding to DNA.

The transcription factor CTCF is involved in many processes, such as embryonic development, the X chromosome inactivation in females, the regulation of the gene cluster recombination during the maturation of immunoglobulin genes, and the regulation of alternative splicing [34-37, 49-51]. CTCF was shown to interact with a large number of proteins (*Fig. 3A*), such as Smad [52], the core transcription factors TFII-I [53] and TAF-3 [54], the helicase p68 containing a DEAD-box domain [55], nucleophosmin, Kaiso [56], TFs YB1, YY1, and Oct4 [57-59], the CHD8 helicase [60], Su(z)12 (polycomb repressive complex 2 (PRC2) component) [61], the deacetylase complex component Sin3A [62], CENP-E [48], and many other proteins [49]. In most cases, individual C2H2 domains of CTCF participate in protein-protein interactions [49]. Probably, CTCF involvement in various processes (the activation and repression of transcription, the long-distance interactions, and TAD formation) is largely reliant on the formation of alternative complexes with partner proteins.

There is experimental evidence demonstrating that CTCF binds to numerous RNAs that modulate its activity. The RNA-binding domain of CTCF combines a portion of the C-terminal domain and two C2H2 domains (10 and 11), non-specifically recognizing RNA *in vitro *[63, 64]. It was suggested that interaction with some RNAs can increase the CTCF ability to form multimeric complexes [63] or to reduce the stability of CTCF binding to DNA [11]. The CTCF activity is also regulated by various posttranslational modifications: poly(ADP)-ribosylation [65], phosphorylation [66], and sumoylation [67].

CTCF has been thoroughly studied and is an example of a TF containing a cluster of C2H2 domains and the unstructured N- and C-terminal regions. The majority of other C2H2 proteins have a similar structure, but their functions and properties have not yet been investigated. It may be assumed that some C2H2 proteins perform functions that are similar to those of CTCF. Interestingly, *Drosophila *mutants in the *ctcf *gene survive to the adult stage, which suggests that insect genomes contain other transcription factors that substitute for CTCF functions [42].

## TRANSCRIPTION FACTORS WITH A CLUSTER OF C2H2 DOMAINS AND AN N-TERMINAL KRAB DOMAIN

**Fig. 4:**
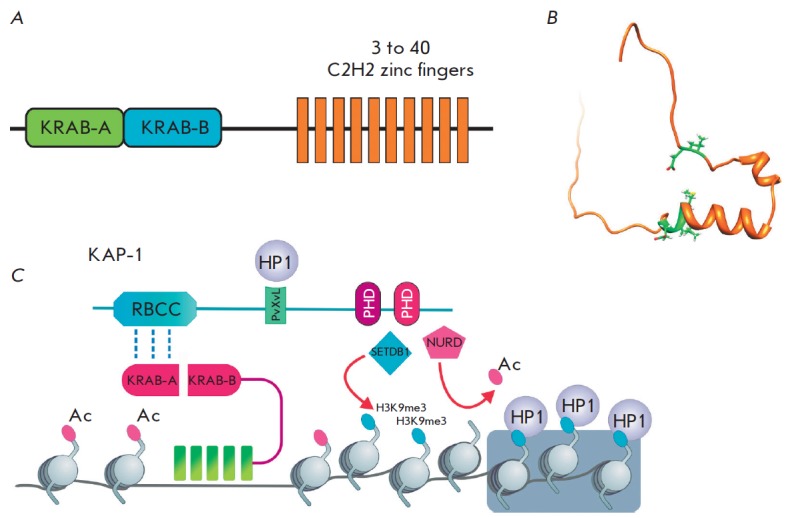
The structure and properties of the KRAB domain. (A) A typical domain structure of the KRAB C2H2 proteins. (B) The NMR structure of KRAB A: 5 mammalian conserved aa are shown in green (DV in positions 6 and 7, and MLE in positions 36–38); they are essential for the KAP-1 recruitment [PDB 1V65]. (C) The mechanism of KAP 1 recruitment and the subsequent formation of the repressive complex.

About one-third of the human proteins with a cluster of C2H2 domains contain the Krüppel-associated box (KRAB) domain at the N-terminus (*Fig. 4A*) [68]. In total, there are 742 different human C2H2 proteins with the KRAB domain, which are encoded by 423 genes [69]. In this case, 384 genes are grouped into 25 chromosomal clusters and only 39 KRAB C2H2 proteins are encoded by single genes. KRAB domain proteins have been found only in tetrapods. The clustering on chromosomes and expansion within large taxa suggest that this family of genes originated through duplications that were preserved by evolutionary selection [70]. The KRAB domain consists of approximately 75 aa and may be structurally divided into two subdomains, A and B, that fold, as predicted, into two amphipathic α-helices (*Fig. 4B*). The KRAB A and KRAB B subdomains are always encoded by separate exons. Alternative splicing produces mRNAs that encode either only the KRAB A subdomain or simultaneously both subdomains, KRAB A and KRAB B, separated by a variable length spacer. Human KRAB proteins can contain from 2 up to 40 C2H2 domains. Unlike genes of other families, the C2H2 domains of KRAB proteins are most often encoded by one exon [71].

The KRAB C2H2 proteins are widely represented in the genomes of tetrapods, and many proteins are involved in the repression of transcription [70]. The versatile and well-studied mechanism of repression is associated with the recruitment of the KRAB-associated protein 1 (KAP-1), which is the only described cofactor of all studied KRAB proteins that represses transcription. The KRAB A domain directly interacts with KAP-1 that, in turn, serves as a platform for recruitment of the repressive complexes (*Fig. 4B*). The five amino acids (*Fig. 4B*) conserved in all the mammalian KRAB A domains (DV, at positions 6 and 7, and MLE, at positions 36–38) are needed for KAP-1 binding [72, 73]. The functional role of the KRAB B subdomain remains unexplored. It has been suggested that this domain increases the efficiency of recruitment of the KAP-1-dependent repressive complex [74]. At the N-terminus of KAP-1, there is the Ring finger/B box/coiled-coil (RBCC) domain that enables binding of KAP-1 to the KRAB domain. The central part of KAP-1 contains a hydrophobic pentapeptide that interacts with the Chromo-Shadow (CS) domain of the HP1 protein. At the C-terminus of the protein, there are two PHD domains that recruit the complexes involved in the deacetylation (NURD) and methylation (SETDB1) of histones [70, 75-77]. The repression initiated at the KRAB C2H2 protein binding site can spread tens of thousands of nucleotides across the surrounding regions of the genome through the successive introduction of the H3K9me3 modification and the subsequent binding to it of the HP1 repressor [78-80]. The KAP-1 expression peak is at the early embryonic stages, and the transcriptional repression by KRAB C2H2 proteins is critical for early embryonic development. At later stages, the somatic cell repression can be maintained by epigenetic mechanisms, with no direct involvement of the KRAB C2H2 proteins [81, 82].

The majority of the KRAB C2H2 proteins are species- and genus-specific. In some vertebrates, such as birds, lizards, and frogs, the KRAB A domain has the multiple amino acid substitutions required for the interaction with KAP-1 [31, 72]. This may be explained either by the fact that the KRAB domain in these vertebrates performs other functions or by the fact that these classes of vertebrates have a modified KAP-1 that retains its ability to interact with the KRAB domain. In general, the evolutionary analysis of the conservatism of KRAB C2H2 proteins has demonstrated that KRAB C2H2 gene families formed independently in each class of vertebrates, which confirms the high evolutionary emergence rate of new genes of this class.

There are only three known KRAB C2H2 genes that belong to a single cluster and are common to all studied vertebrate species. These genes encode C2H2 proteins containing a structurally modified KRAB domain that no longer binds to the KAP-1 repressor and is involved in transcriptional stimulation [31, 83, 84]. Of particular interest is the highly conserved gene *Meisetz *(PRDM9) that codes for not only a modified KRAB domain, but also the SET domain [85, 86]. The SET and KRAB domains jointly recruit the H3K4-methyltransferase that is responsible for trimethylation of histone H3 on lysine 4 (H3K4me3). The H3K4me3 modification at the promoter region usually correlates with an active transcription. A bioinformatic analysis demonstrated that a portion of the KRAB domain encoded by the *Meisetz *gene is homologous to the KRI motif present in the genomes of all well-studied eukaryotes, including arabidopsis, rice, fungi, and yeast [31, 86]. The widespread occurrence of the KRI motif suggests that the KRAB domain of the Meisetz protein might have originated from this motif by addition of several amino acid residues. The KRAB domain might have acquired its repressor functions through random mutations that allowed the repressor to bind to KAP-1, which was preserved during evolution.

It was experimentally demonstrated that TFs of the KRAB C2H2 class in vertebrates play an important role in various processes of embryonic development, cell differentiation and proliferation, and the regulation of the cell cycle and apoptosis [70, 73]. The binding sites for KRAB C2H2 proteins correlate with the open (nucleosome free) chromatin regions, something that is explained by the binding with the active regulatory regions of the genes [87]. Whole-genome studies have demonstrated that KRAB C2H2 proteins bind to the enhancers and promoters of genes and can activate transcription in some cases [88-90]. The ability of KRAB C2H2 proteins to activate transcription should correlate with the suppression of interaction between the KRAB domain and KAP-1. The mechanism of this suppression still remains unexplored but is probably associated with reversible modifications of the amino acid residues of the KRAB domain. An important role may be played by the C2H2 domains that are potentially capable of recruiting individual TFs and whole complexes that positively/negatively regulate transcription.

The above-mentioned Meisetz protein (PRDM9), which is expressed only in mammalian gonads, plays an interesting role [91]. Most mammalian recombination hotspots were found to contain a potential PRDM9 binding site [92]. Rapid evolutionary changes in the number and primary structure of C2H2 domains led to the binding of PRDM9 to different nucleotide DNA sequences in different mammals [91, 93-96]. The binding of PRDM9 results in the formation of a nucleosome-depleted region and H3K4me3 modification of the surrounding nucleosomes [97]. The SPO11 complex inducing double-strand breaks is supposed to simultaneously recognize the histone H3K4me3 mark and directly bind to PRDM9 [98].

Recently, a new functional role played by KRAB C2H2 proteins in the repression of foreign DNA transcription, primarily, of endogenous retroviruses and the mobile elements LINE and SINE, was discovered [79, 87, 99, 100]. Mobile elements constitute a significant part of the mammalian genome, and repression of their transcription is essential [101]. Different KRAB C2H2 proteins bind to the regulatory regions of mobile elements and those of certain retroviruses, and they induce their epigenetic repression. There is a hypothesis that holds that the newly appeared KRAB C2H2 proteins have been preserved by evolutionary selection, because they play a critical role in the suppression of the expression of new mobile elements, while the more conserved KRAB C2H2 proteins participate in the regulation of the expression of cellular genes [79].

Another explanation for the rapid evolution of the genes that encode KRAB C2H2 proteins may be their key role in the control of the expression of the genes that determine the development of the nervous [102] and circulatory [103] systems of mammals. For example, many genes encoding the KRAB C2H2 proteins specific to humans and primates are actively transcribed in the brain [102]. However, there is no direct correlation between the number of KRAB C2H2 genes and the level of organism complexity. For example, the number of KRAB C2H2 genes in opossum is double that in humans [31]. It is hoped that the emergence of new technologies for specific antibody generation, whole-genome analysis, and single gene mutations using the CRISPR/Cas9 system will soon clarify the functional role played by KRAB C2H2 proteins.

## TRANSCRIPTION FACTORS WITH A CLUSTER OF C2H2 DOMAINS AND AN N-TERMINAL SCAN DOMAIN

**Fig. 5:**
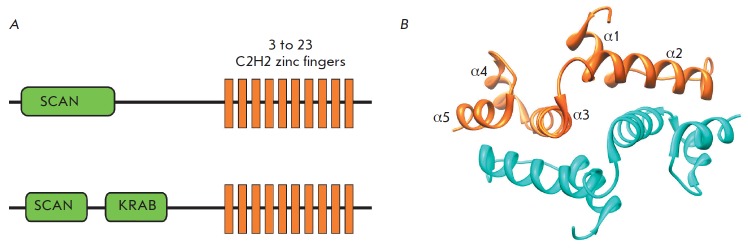
The structure and properties of the SCAN domain. (A) A typical domain structure of the SCAN C2H2 and SCAN KRAB C2H2 proteins. (B) The crystal structure of a SCAN domain dimer from the Zfp206 protein [110].

The SCAN domain was first described in human ZNF174 TF [114] (*Fig. 5A*). Subsequently, proteins with these domains were found in some other classes of vertebrates [104]. In humans, mice, and cows, 71, 38, and 28 SCAN C2H2 proteins were found, respectively [94]. Genes encoding the SCAN proteins usually occur in the genome as small (two to seven) clusters [104]. The SCAN domains in clusters have a higher degree of homology among themselves, which suggests that they emerged through gene duplication and a subsequent adaptive evolution. Approximately half of the genes encode simultaneously the SCAN and KRAB domains and usually occur in large clusters, along with the genes encoding only the KRAB C2H2 proteins (*Fig. 5A*) [27, 94].

The SCAN domain structure is highly similar to that of the C-terminal domains of the human immunodeficiency virus capsid protein [105] and the Gag protein from the family of *Ty3/gypsy *retrotransposons [27]. Based on such data, it was suggested that SCAN domains initially originated from the retrovirus capsid proteins in the lower vertebrates; then, during evolution, this domain acquired a new function in TFs-containing clusters of the C2H2 domains [106]. The KRAB domain in combination with the SCAN domain is present in mammals and lizards, but it is absent in chicken and frog.

To date, the spatial structures of the SCAN domains of the proteins Zfp206 [107], PEG3 [108], ZNF24, ZNF174 [105], and MZF-1 [109], which have a high degree of homology, have been resolved (*Fig. 5B*). The features of the spatial structure may be illustrated by the example of the SCAN domain of Zfp206 [107], which exists as an antiparallel homodimer. Each monomer in the homodimer consists of five α-helices. The core of the inner homodimer surface is formed by packing of the second α-helix of one monomer against the third and fifth α-helices of the opposing monomer and *vice versa*. The N-terminal first α-helix provides additional contacts of one monomer with the third α-helix of the opposing monomer (*Fig. 5B*). All SCAN domains can form homodimers, but only some SCAN domains are able to form heterodimers [104, 111-113]. The first α-helix of the SCAN domain has the greatest variability in the hydrophobic amino acid sequence and is considered as a potential candidate that determines the formation of heterodimers from different SCAN domains. For example, the SCAN domain of Zfp206 was shown to be able to form a heterodimer with a similar domain in Zfp110 [107]. The replacement of the first α-helix with an α-helix of a heterologous SCAN domain of ZNF174 or its removal results in a loss of the ability of these SCAN domains to form heterodimers.

The CRAB A domain is known to recruit the repressive complex, whereas there is no evidence of SCAN domain effect on transcription [104, 111]. There is only fragmentary data on the functional role of SCAN C2H2 TFs. For example, human ZNF263 TF containing the SCAN and KRAB domains and 9 C2H2 domains predominantly binds to the promoter regions and is able to participate in both the activation and repression of transcription [89]. Another member of the family, the ZNF658 protein, also contains the SCAN and KRAB domains and is involved in the activation of the expression of the rRNA genes that are transcribed by RNA polymerase I [115]. Probably, the main function of SCAN proteins may be related to their ability to form homo- and heterodimers between the SCAN domains.

## TRANSCRIPTION FACTORS WITH A CLUSTER OF C2H2 DOMAINS AND AN N-TERMINAL ZINC FINGER-ASSOCIATED DOMAIN

**Fig. 6:**
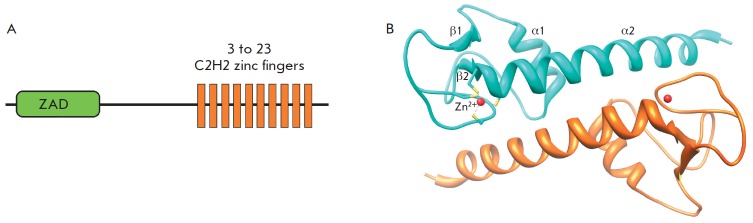
The structure and properties of the ZAD. (A) A typical domain structure of ZAD C2H2 proteins. (B) The crystal structure of a ZAD dimer from the Grau protein [117].

The zinc finger-associated domain (ZAD) (*Fig. 6A*) is found at the N-terminus of the C2H2 proteins of many arthropods [28]. In vertebrates, only one protein containing an N-terminal structure similar to the ZAD has been found [116]. In the genomes of *Anopheles gambiae*, *Drosophila melanogaster*, and *Apis mellifera *(honey bee), the 147, 98, and 29 ZAD C2H2 proteins, respectively, were found [116], whereas only four genes encoding ZAD-like domains were found in crustaceans (*Daphnia pulex*). Usually, genes encoding highly homologous ZADs form small clusters. It is suggested that these genes originated from multiple duplications of original copies and then were preserved through positive selection [28, 116]. Probably, the evolutionary process was very fast, since obvious homologs were found only for a few ZAD C2H2 proteins in distant *Drosophila *species [116].

The ZAD size varies between 71 and 97 aa. The multiple alignments of the sequences of 32 family members demonstrate that this domain consists of four conserved blocks linked by regions of varying lengths [28]. A distinctive feature of ZADs is the presence of two invariant cysteine pairs coordinating a zinc ion.

To date, the crystal structure of only one ZAD from the Grauzone protein (Grau) has been resolved [117], which can serve as a prototype for all ZAD structures. The N-terminal ZAD portion forms a globule around the zinc ion, and the C-terminal stem is formed by a long α-helix 2 (α2) that comprises almost one-third of all the amino acids in the ZAD. The ZAD folding largely depends on the coordination of two cysteine pairs (separated by about 50 aa) by the zinc ion, which results in drawing of the β2-α2 regions and the N-terminus to the domain center.

In a crystal, two ZAD molecules are associated as an antiparallel dimer. Most of the amino acid residues that are conserved in the ZAD family [28] form contacts between the two subunits. The ZAD of Grau has a negatively charged surface, suggesting that the domain is unable to bind to DNA [117]. It has been suggested that the main function of the ZAD is to form homodimers of the C2H2 proteins [118]. ZADs also participate in the regulation of the nuclear localization of some of the ZAD C2H2 proteins [119].

Proteins with ZADs account for approximately one-third of the total number of proteins with C2H2 clusters and one-tenth of all TFs in the *D. melanogaster *genome [28]. To date, the functions of only a small fraction of ZAD C2H2 TFs have been studied. The majority of ZAD C2H2 proteins are expressed during oogenesis and at early embryogenesis [116]. The results of several studies point to an important functional role for ZAD C2H2 proteins in *Drosophila *development.

The Motif 1 Binding Protein (M1BP) is expressed at a high level in all tissues and at all stages of *Drosophila *development and is a key factor in the organization of the architecture of more than 2,000 *Drosophila *promoters with a characteristic motif (T/C)GG(T/C)CACACTG [120].

In transgenic *Drosophila *lines, three ZAD C2H2 proteins (Pita, ZIPIC, and Zw5) exhibit the properties of insulator/architectural proteins: they block the interaction between an enhancer and a promoter and maintain long-distance interactions [118, 121-124]. The ZADs of these proteins form only stable homodimers [118]. Interestingly, the DNA fragments containing binding sites for different ZAD C2H2 proteins cannot maintain long-distance interactions, which suggests a key role for the ZAD dimerization in the formation of specific contacts between distant chromatin regions. Indeed, the ZAD of ZIPIC is required for the maintenance of long-distance interactions between the GAL4 activator and the reporter gene promoter in yeast *Saccharomyces cerevisiae *[118]. As in the case of M1BP, binding sites for the proteins ZIPIC, Pita, and Zw5 are predominantly located close to the transcription starts [118, 125], which suggests an architectural function for these proteins in the promoter organization. Null mutations in the genes encoding the Pita and Zw5 proteins lead to late embryonic and early larval lethality, which indicates that there is an important role for these proteins in early *Drosophila *development [121, 126].

The Grau protein is expressed at all stages of *Drosophila *development; it is found in the nuclei of the nurse and follicular cells surrounding the oocyte [127, 128]. Mutations in this gene lead to oogenesis arrest at the meiosis II stage, which is related to the role of Grau in the activation of the promoter of the *cortex *gene that regulates meiosis in oocytes [127, 128]. The Serendipity delta protein (Sry δ) binds to the promoter of the *bicoid *gene, which plays a key role in early embryogenesis, and stimulates transcription of the gene [129]. Null mutations in the *sry δ *gene manifest themselves as embryonic lethals, which indicates the significance of Sry δ in the early development of *Drosophila *[130].

The Trade Embargo protein (Trem) is expressed mainly in *Drosophila *germ cells and probably performs a function similar to that of the PRDM9 protein in mammals [95, 97, 131]. Trem specifies the binding sites for the Mei-P22 protein that is involved in the induction of meiotic chromosomal breaks [131]. Mei-P22 and its partner, Mei-W68, participate in the formation of the double-strand breaks that initiate crossing-over in meiosis [132-134]. According to the model, Trem, to gether with its partners, creates the open chromatin regions that recruit, through specific protein-protein interactions, the Mei-P22/Mei-W68 complex, inducing double-strand breaks [131].

In general, the available data demonstrate that ZAD C2H2 TFs play an important role in the organization of the structure and functional activity of promoters, the recruitment of protein complexes, and the formation of the chromosomal architecture.

## CONCLUSION

At present, there are many unresolved issues related to the regulation of gene transcription, the organization of the structure of regulatory elements, and the mechanisms of long-distance interactions. It is also quite obvious that the vertebrate CTCF cannot be the sole and key DNA-binding protein that determines the architecture of vertebrate chromosomes [7].

Unlike the well-studied TFs of other classes, C2H2 proteins specifically bind to long DNA sequences reaching several tens of base pairs. The C2H2 proteins can effectively bind to DNA as monomers, unlike most other TFs that bind to short palindromic sequences as homo- or heterodimers. Some C2H2 domains in combination with the unstructured regions of C2H2 proteins can enable a variety of interactions with protein complexes and individual TFs and RNAs. Therefore, C2H2 proteins may be considered as promising candidates for the role of organizers of the architecture of regulatory elements, such as promoters, enhancers, insulators, and silencers. Unfortunately, the available experimental evidence is insufficient in order to confirm the validity of this assumption for vertebrates. On the other hand, the well-studied CTCF protein of vertebrates has a number of properties (the specific binding to a DNA site, the formation of open chromatin regions, the recruitment of protein complexes, and the organization of long-distance interactions) that may be extrapolated to other C2H2 proteins.

Finally, many C2H2 proteins have domains that are capable of homodimerization. Interestingly, in arthropods and vertebrates, there was an expansion of different domains: ZAD and SCAN, respectively. The main common property of the ZAD and SCAN domains is their ability to preferentially form homodimers. Homodimerizing ZADs of the three ZAD C2H2 proteins (Pita, ZIPIC, and Zw5) were demonstrated to determine the specificity of long-distance interactions [118]. Probably, other ZAD C2H2 proteins possess similar properties. So far, only some data on the role of SCAN C2H2 proteins in the organization of active promoters in vertebrates has been obtained. Apart from the ZAD and SCAN domains, C2H2 proteins may have other domains capable of multimerization: e.g., an N-terminal domain of the *Drosophila *dCTCF protein [42].

Therefore, the available fragmentary data already allow us to suggest a model where the C2H2 proteins act as the messengers in the transfer of information from the nucleotide sequence of the regulatory elements (promoters, enhancers, and silencers) to the protein complexes that determine the properties of regulatory elements. It is assumed that investigation of individual members of this extensive class of TFs, the elucidation of the functional roles of the ZAD, SCAN, and KRAB domains, and the identification of new partner proteins and new dimerization domains will allow us to evaluate the real contribution of C2H2 proteins to the formation of the chromosomal architecture and the structure of regulatory elements.

## Abbreviations

aa: amino acid
bp: base pair
C2H2 proteins: proteins containing clusters of C2H2 zinc fingers
TAD: topologically associated domain
TF: transcription factor
